# Chemosignals of Stress Influence Social Judgments

**DOI:** 10.1371/journal.pone.0077144

**Published:** 2013-10-09

**Authors:** Pamela Dalton, Christopher Mauté, Cristina Jaén, Tamika Wilson

**Affiliations:** Monell Chemical Senses Center, Philadelphia, Pennsylvania, United States of America; Medical University of Vienna, Austria

## Abstract

Human body odors have important communicative functions regarding genetic identity, immune fitness and general health, but an expanding body of research suggests they can also communicate information about an individual’s emotional state. In the current study, we tested whether axillary odors obtained from women experiencing psychosocial stress could negatively influence personality judgments of warmth and competence made about other women depicted in video scenarios. 44 female donors provided three types of sweat samples: untreated exercise sweat, untreated stress sweat and treated stress sweat. After a ‘washout’ period, a commercial unscented anti-perspirant product was applied to the left axilla only to evaluate whether ‘blocking’ the stress signal would improve the social evaluations. A separate group of male and female evaluators (n = 120) rated the women in the videos while smelling one of the three types of sweat samples. Women in the video scenes were rated as being more stressed by both men and women when smelling the untreated vs. treated stress sweat. For men only, the women in the videos were rated as less confident, trustworthy and competent when smelling both the untreated stress and exercise sweat in contrast to the treated stress sweat. Women’s social judgments were unaffected by sniffing the pads. The results have implications for influencing multiple types of professional and personal social interactions and impression management and extend our understanding of the social communicative function of body odors.

## Introduction

The ability of human body odor to communicate information between individuals is supported by an ever-expanding body of research. Not only have body odor signals been shown to convey messages about genetic relatedness, mating fitness and general health [1], [2], but body odors produced from individuals in specific emotional states have been shown to modify both the neural and behavioral states of the receiver, whether or not they are consciously aware of the source or nature of the body odor [3]–[6]. In studies designed to explore the functional implications of emotional chemosignals, it has been shown that smelling sweat produced from stressed individuals potentiates the human startle reflex [7], improves the discrimination of fearful [8] and angry [6] faces, elicits empathy [9] and enhances vigilance and attention [7], [10]–[12]. More recently, de Groot et al, tested the ability of emotional chemosignals to recruit joint processes between the sender and receiver and found that inhaling chemosignals emitted during emotional states induced the same state (fear, disgust) in the receiver [13], consistent with theories of emotional contagion [14].

To further investigate the social communicative function of body odors, we chose to evaluate the degree to which smelling body odors from a stressed individual would alter one’s perception of another individual, specifically with regard to social judgments of their traits and evaluations of emotional states. The dual-dimension theory of social judgment has guided research that reliably demonstrates that warmth (friendliness, sincerity & trustworthiness) and competence (intelligence, efficacy & confidence) are the fundamental universal dimensions on which others are judged [15] and account for as much as 82% of the variance in perceptions of everyday social behaviors [16]. People perceived as warm and confident elicit uniformly positive responses while those perceived as lacking warmth and competence are judged negatively. From spontaneous impressions of presidential candidates to evaluations of job candidates, such impressions can dramatically alter liking and acceptability. Chemosensory signals of stress have the potential to communicate social impressions of an individual which may be translated into poorer judgments of their competence and warmth. The goal of the current study was to evaluate how stress odors collected from donors would impact social judgments of women depicted in videos relative to body odors collected from those same donors during exercise. We also evaluated whether ‘blocking’ the stress signal with a commercial, unscented, anti-perspirant product would improve the social evaluations.

The Trier Social Stress test (TSST) is the most validated and effective protocol for inducing moderate levels of psychosocial stress in a laboratory setting [17]. The TSST requires subjects to give an impromptu speech and to perform mental arithmetic before a set of judges. The TSST has been shown to activate the two major stress pathways, the hypothalamic-pituitary-adrenal axis (HPA) and the sympathetic-adrenal-medullary axis, resulting in increases in heart rate, skin conductance and endocrine stress markers such as cortisol in serum and saliva [18]. Axillary secretions produced in response to stress have been shown to contain more odiferous compounds due to the release of apocrine gland secretions, relative to axillary secretions produced from eccrine glands in response to exercise [19].

## Materials and Methods

### Donor Participants

Sweat samples were obtained from the axillary area of 44 female donors, aged 18-45, mean  = 30.32, SEM  = 1.34). All donors participated in two conditions: a ‘stress’ condition in which they were administered the TSST and 30 minutes later, an ‘exercise’ condition in which they cycled on a stationary cycle for the same amount of time as the TSST (15 minutes). For the stress condition, samples were obtained from both underarms, one of which was untreated with any deodorant or anti-perspirant product, the other which was treated with a commercially-available antiperspirant product for 5 days prior to the test. For the exercise session, only the untreated underarm was sampled.

### Evaluator Participants

The effects of the donor sweat samples were tested on a different group of 120 Evaluator participants, aged 18-55 (Males, n = 48: mean age = 29.6, SEM = 1.33; Females, n = 72: mean age =  32.2, SEM = 1.14). Evaluators sniffed the three types of sweat (exercise, untreated stress and treated stress), blinded to condition, while viewing and rating a series of videos.

In order to understand whether any effects were due to differences in the odor impact of the different sweat samples, we recruited a separate group of 10 females to evaluate the three types of donor pads for intensity and pleasantness.

### Ethics Statement

The study was reviewed and approved by the University of Pennsylvania Institutional Review Board and all participants provided written informed consent.

### Donor Procedure

Sweat samples were obtained from the Donors using procedures to maintain the integrity of the samples and to prevent contamination by other odors. Briefly, donors engaged in a 15 day washout period in which they were instructed to wash each day, paying particular attention to the underarm area, with the supplied non-fragranced body wash and shampoo. The participants were asked to refrain from using any fragranced product, including perfumes, lotions, and oils throughout this period and to avoid odorous food and alcohol consumption on the three days prior to their test session.To facilitate compliance with the washout regimen, for the first 7 days the participants were allowed to use a supplied commercially-available deodorant product (Tom’s of Maine™, aluminum-free, non-fragranced deodorant) on each underarm in the mornings. For days 8, 9 and 10, participants were asked to refrain from using any deodorant or antiperspirant in the underarm area. Participants were then instructed to use a commercially-available and supplied antiperspirant product (Secret Clinical Strength™, non-fragranced antiperspirant for sensitive skin) on their left underarm only on the nights of days 11–14, then again 2 hours before their test on Day 15. A calendar was generated for each participant so they could keep track of exactly what to do on each day of the washout period, and the technical staff maintained contact with the participants in case they had questions during this period.

**Figure 1:**
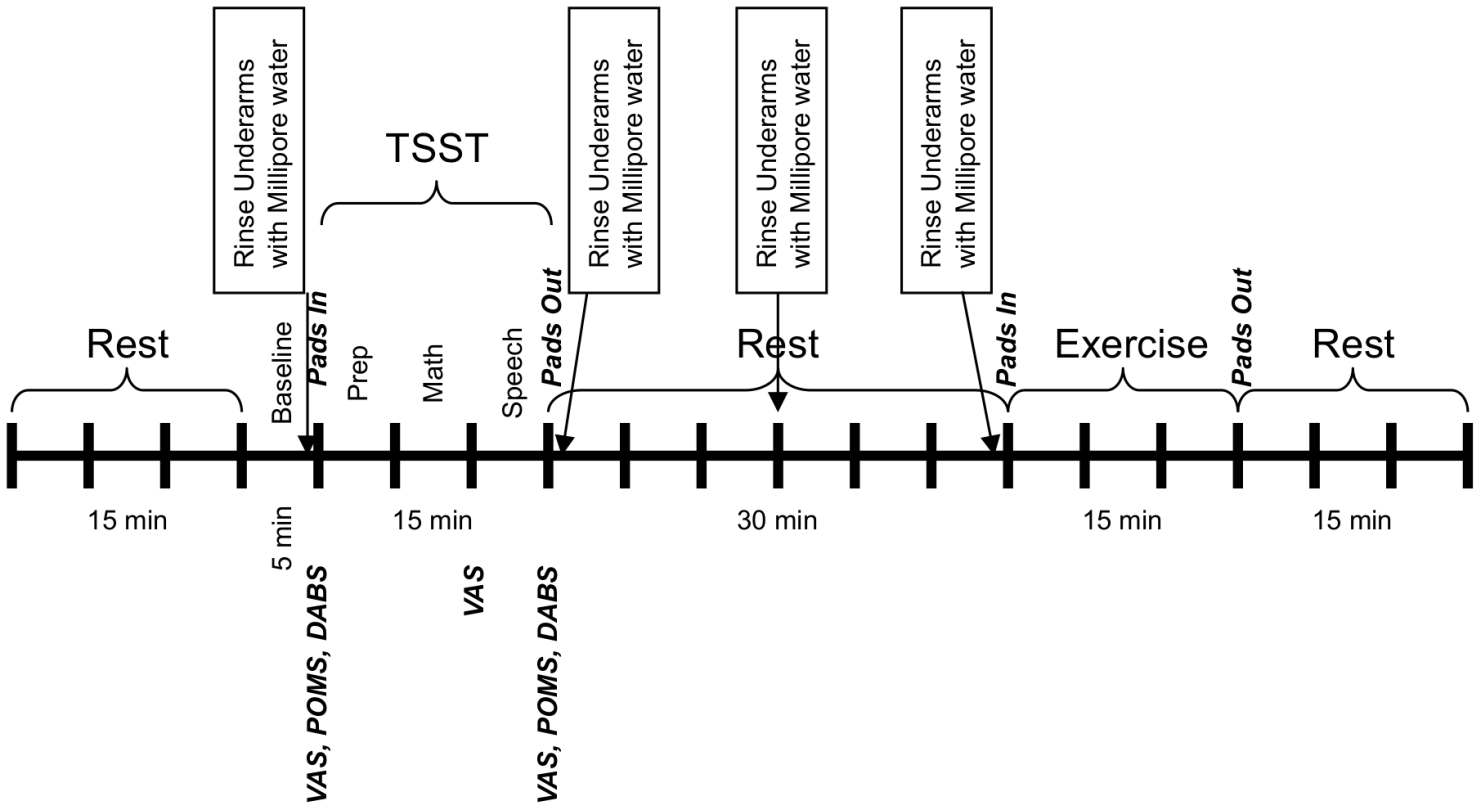
Timeline of events and measures for the donor session.

On the test day, participants came to the Center wearing comfortable clothes for exercising that also would allow access to the underarm area for sample collection. On each day, successive donors were tested at either 9 am or 1 pm. The timeline of events is shown in Figure 1. They were seated in a climate-controlled chamber and baseline recording of heart rate and self-reports of current mood were obtained. After rinsing the underarm area with Millipore water and drying it, clean 4 inch square cotton pads were then placed in both axillary areas for sweat sample collection.

A different experimenter then entered the room to administer the TSST, which was comprised of 5 minutes of speech preparation, 5 minutes of mental arithmetic and 5 minutes of public speaking. After the TSST, the participants again filled out the mood ratings. Following that, the cotton pads were removed and the untreated (right) axillary area was rinsed again.

Over the next 30 minutes, the participant was debriefed about the misinformation regarding evaluation of their performance during the TSST and informed that they would be asked to exercise on a stationary cycle for 15 minutes. Their right axillary area was again rinsed with Millipore water.

For the exercise session, the donor was escorted into another testing room with a stationary exercise bicycle. A cotton pad was placed under the untreated (right) axillary area and the participant was asked to cycle to a pre-determined individual target heart rate, for 15 minutes, whereupon the pad was removed.

### Odor Stimuli and Delivery

Sweat pads obtained from each successive group of three donors (a total of 15 donor groups) were cut into four pieces and four ‘pooled’ samples of each of the three types of sweat (untreated exercise sweat, untreated stress sweat and treated stress sweat) were constructed. Pads were immediately frozen after collection until the evaluation day at –80°C in glass containers to minimize contamination or bacterial growth which can produce additional odors. A custom-built air-dilution olfactometer with 6 channels was used to deliver each type of odor plus clean air to the evaluators through a nasal cannula that directed the flow of odorant (at a rate of 2.5 liters/minute) into the participant’s nostrils. To reduce potential biasing effects of inter-individual variability in sweat production, each successive eight evaluators were tested with pads from the same three donors collected across the three sweat conditions. For example, participants 1–8 were tested with untreated, treated and exercise sweat pooled from donors 1–3, participants 9–16 were tested with untreated, treated and exercise sweat pooled from donors 4–6, and so on.

### Videos

**Figure 2:**
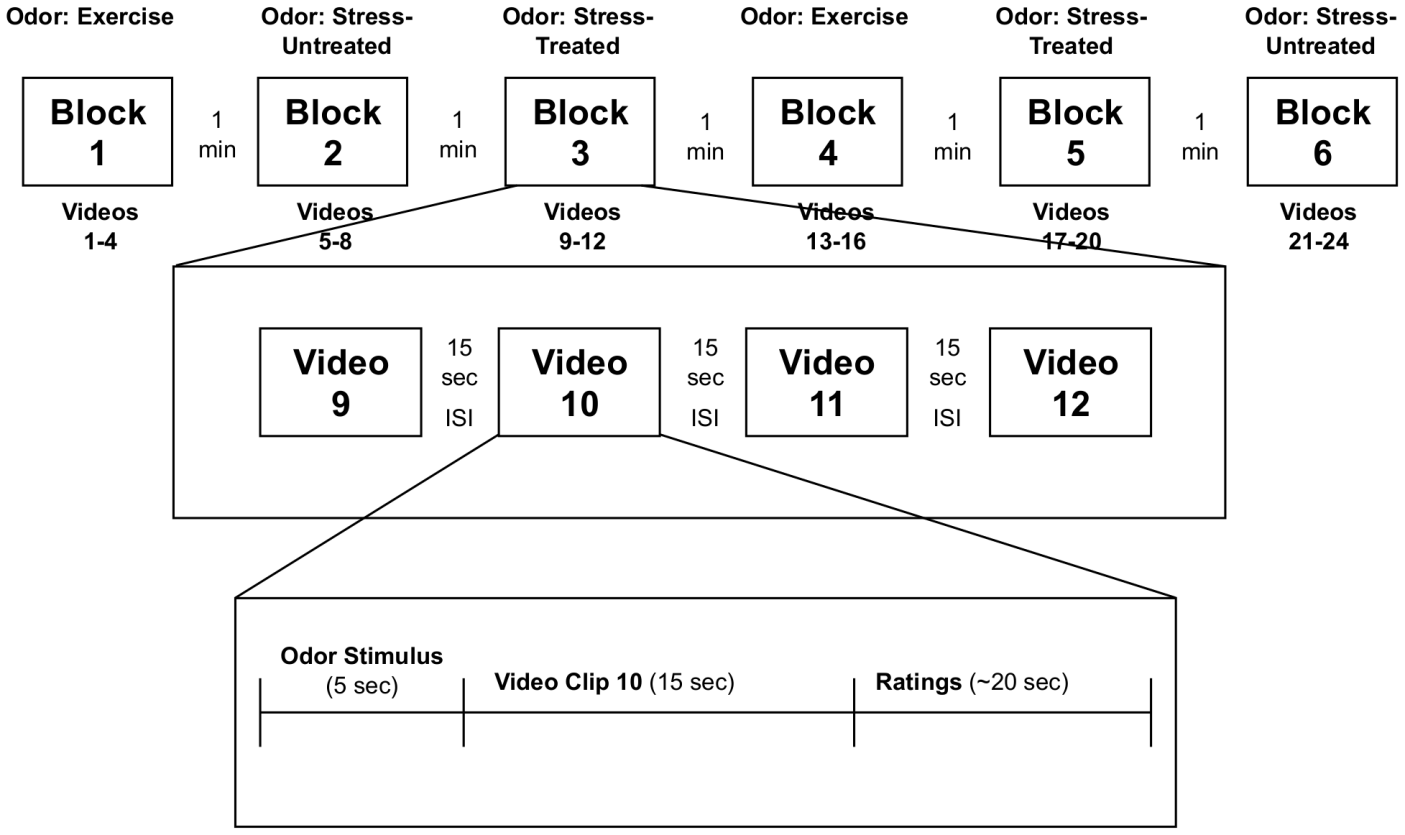
Schematic of the study design for presenting stimuli and collecting data. Across subjects, blocks were counterbalanced so that each odor stimulus was paired with each video twice.

24 different videos were presented for ratings. Videos of females interacting (without audio) in everyday situations (office, home, child care) were obtained from Getty Images (http://www.gettyimages.com/). The initial search generated 90 video clips which were each rated for the emotional state of the female and the stress level of the situation on a 7 point scale (1 =  very relaxed; 4 =  neutral; 7 =  very stressed) by 8 judges (3 males; 5 females). The videos with an average rating of 3.5 to 4.5 were determined the ‘most neutral’ out of the initial 90 video clips. This first cut left us with 39 video clips. After removing the redundant video clips (clips that were different versions of the same person doing the same thing), we were left with 24 video clips that we used in the study. We then converted the video clips from Quicktime and imported them into Microsoft Windows Movie Maker (Version 5.1) for editing. Only one activity was depicted in each video and each lasted for 15 seconds. Videos were presented in blocks of four and for a single participant each block was paired with only a single type of sweat odor. However, each block of four videos was counter-balanced over participants such that each set of 4 videos was presented with each sweat odor condition (Figure 2).

### Evaluator Procedure

On the day of testing, participants provided informed consent and filled out other paperwork (demographics, health screening, personality questionnaires and chemical sensitivity scale). Once paperwork was completed, the participant was asked to adjust a chin rest on the table to a comfortable height, and the nose-pieces attached to the ports of the olfactometer were adjusted to fit comfortably into the nostrils of the participant.

A practice script was then begun to acclimate the participant to the routine of the task they were to perform, which had 4 steps: 1) they were instructed to pay attention to a woman in the upcoming video, 2) they were instructed to inhale the odor stimulus, 3) a 15-second video clip was presented immediately after the odor stimulus, and 4) they were asked to make ratings of confidence, stress, trustworthiness and competence about the women in the video that was just presented. Ratings were made on the computer by positioning a slider along a visual analog scale that ranged from ‘Not at All’ to ‘Extremely’ for each of the judgments. All of these steps, including the syncing of the odor stimulus with the olfactometer, were programmed into ePrime software (Psychological Software Tools, Inc., Sharpsburg, PA) and presented twice for the sake of practice.

After the practice script was completed (from which none of the data were collected), the participant began the study. Depending on the counterbalanced group they were in, the blocks of videos were presented in one of six different orders. Between each video in a block, there was a 15 second inter-stimulus interval, and between each block there was a 60 second break during which the participant could remove him/herself from the apparatus and stretch or relax until they were cued for the next block of videos. Based on recent literature [13], a 60-second interval between successive sweat conditions was deemed adequate for differential emotion elicitation. After completing 6 blocks of ratings, the participants were debriefed and dismissed.

### Statistical Analysis

The data were analyzed using Analysis of Variance (ANOVA) and Bonferroni post-hoc tests (Statistica, 9.0, Statsoft, Inc. Tulsa, OK.). Significance was evaluated at p<0.05 unless otherwise noted. To ensure that the stress manipulation was effective for the Donors, both objective (Heart Rate) and subjective (Visual Analog Scale [VAS] Ratings, Profile of Mood State [POMS] Scores) endpoints were analyzed using Time (Baseline vs. post-TSST) as the within group variable. For each Evaluator’s rating, the position along the Visual Analog Scale was converted to a number between 0–100 and we calculated an average score for each rating type made within the same odor condition. Thus, each subject had an average rating of Confidence, Trustworthiness, Competence and Stress for women in videos paired with each of the three sweat odor conditions. The data were analyzed using ANOVA with odor condition as the within-group variable and alpha levels for significance for the three personality ratings were adjusted using the Bonferroni-Holm correction for multiple comparisons [20]. Effect sizes are indicated for the major significant findings (Cohen’s *d*).

## Results

### Stress Manipulation

The data from the Donors demonstrated that the TSST effectively elicited a stress response, as evidenced by an increase in heart rate, self-reported stress and anxiety ratings. Heart rate (HR) significantly increased during the TSST (mean = 86.44; SE = 1.43) as compared with baseline (mean = 78.14; SE = 2.36; F_(1,43)_ = 15.70, p<0.001). Ratings of anxiety and stress also increased during and immediately following the TSST (Baseline vs Mid vs Post), (Anxiety VAS: F_(2,86)_ = 12.138,p<0.001; Stress VAS: F_(2,86)_ = 15.401,p<0.001), with significant increases at alltime points from baseline. However, although the ratings of stress and anxiety decreased following the TSST, they did not return to baseline (Anxiety VAS mean/SEM: Pre: 29/3.9, Mid: 53/4.7, Post: 47/4.4 and Stress VAS mean/SEM: Pre: 28/3.7, Mid: 55/4.1, Post: 43/4.0).

The POMS scale contains multiple items that comprised an ‘Anxiety’ score and Anxiety scores increased significantly following the TSST when compared with Baseline scores (POMS Anxiety: F_(1,43)_ = 30.115, p< 0.001).

### Evaluation of Stress Levels in Videos

Participants rated the women in the videos on the dimensions of how stressed they appeared. An Analysis of Variance conducted on the stress ratings revealed a main effect of Axillary Odor: videos paired with untreated stress and exercise sweat elicited higher ratings of stress than did videos paired with the treated stress sweat, F_(2, 236)_ 9.83, p<0.001, Cohen’s *d* = 0.35 and 0.33 for untreated stress vs treated and exercise vs treated stress sweat, respectively. The Mean/SEM stress ratings were 48.2/1.47, 47.5/1.44 and 42.4/1.37 for videos paired with exercise, untreated stress and treated stress sweat, respectively. Comparing the responses to the videos across genders, we found no differences in the ratings as a function of the type of odor they were paired with, F_(1,118)_  = 1.23, p>0.1. Thus, it appears that with respect to rating the stress levels of the women in the videos, both men and women were equally influenced by the axillary odors from untreated stress samples.

### Social Judgments in Videos

**Figure 3:**
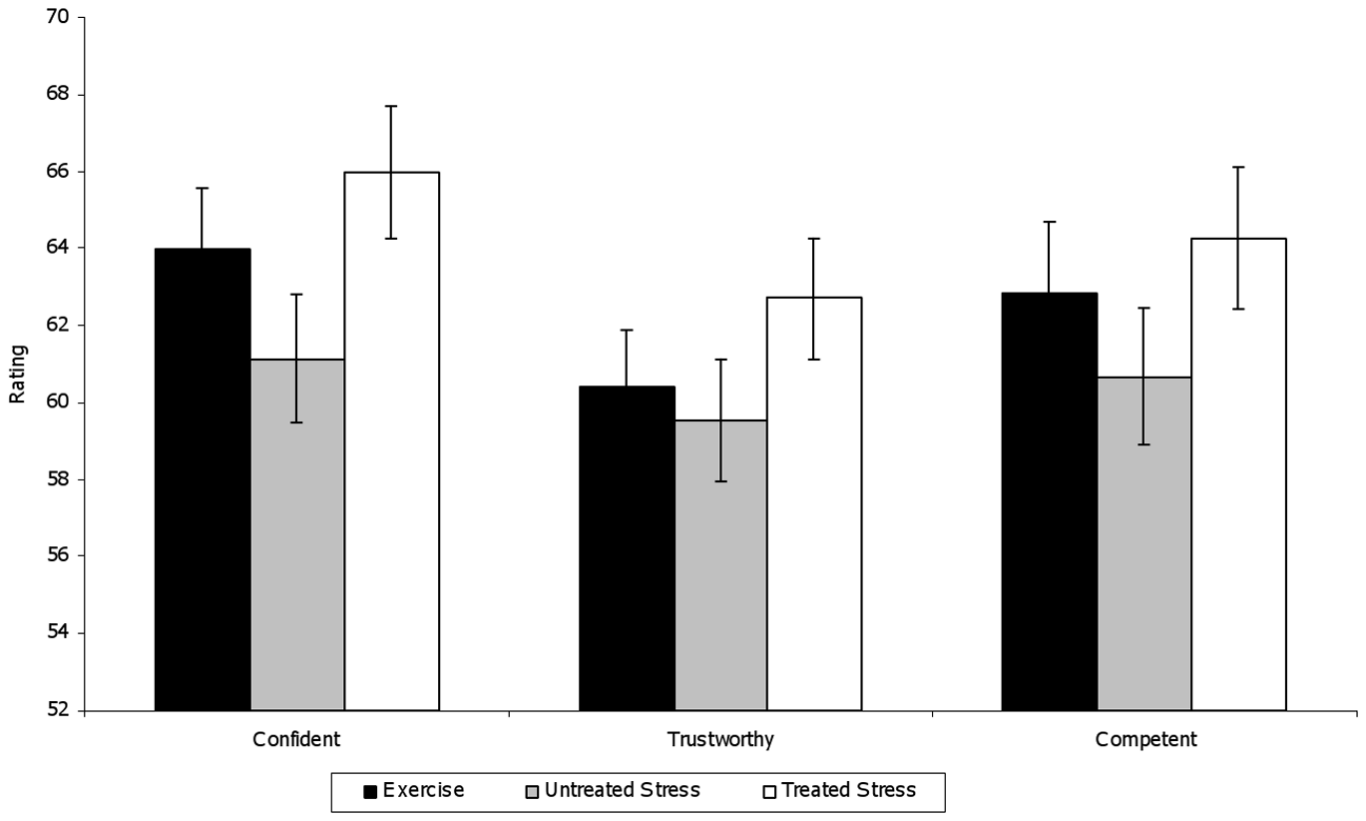
Mean and standard errors of the confidence, trustworthiness and competence ratings of the women depicted in the videos as a function of the sweat odor pairing condition.

We first conducted an ANOVA on the ratings of Competence, Confidence and Trustworthiness for the women in the videos as a function of odor condition and this revealed a main effect of condition, F _(2, 236)_  = 4.74, p = 0.009. Women in the videos who were evaluated in the presence of untreated stress sweat were consistently rated the lowest on these dimensions, yet post hoc tests (Bonferroni) revealed that the differences did not reach significance when compared with women rated in the presence of exercise sweat. However, overall ratings for women evaluated in the presence of the untreated stress sweat were significantly lower than ratings for women evaluated in the presence of treated stress sweat, p < 0.01, Cohen’s *d* = 0.28. As shown in Figure 3, separate ANOVAs on each type of rating as a function of odor condition also confirmed significant differences (Bonferroni-Holm corrected [20]) in social evaluations made in the presence of untreated vs. treated stress sweat (p = 0.017, p =  0.025 and p = 0.048 for confidence, trustworthiness and competence, respectively ).

ANOVAs conducted for the males and females separately further revealed that the main effect of odor condition was largely driven by the social judgments made by the males. Male evaluators rated the women in the videos significantly less confident, competent and trustworthy when the videos were paired with the untreated stress sweat than when they were paired with the treated stress sweat, F_(2, 94)_  = 4.27, p = 0.01; Cohen’s *d* = 0.41. Surprisingly, although females’ ratings of stress in the videos were similar to those of the males, their social judgments of those women did not differ among the three odor conditions, p> 0.1.

### Evaluation of Donor Sweat for Intensity and Pleasantness

Because it was possible that the effects on social judgments were due to the sweat odors, which could be stronger or more negative during the untreated stress condition, we recruited a separate group of 10 female evaluators to rate the three types of donor sweat samples for intensity and pleasantness. Consistent with previous results, [3], there were no significant differences in the rated intensity or pleasantness of the three types of sweat samples, F_(2,18)_  = 0.94, p>.1 and F_(2,18)_  = 0.87, p>.1 for intensity and pleasantness, respectively. The rated intensity for exercise, untreated stress and treated stress sweat was between weak and moderate (Mean/SEM = 26.8/7.7, 25.0/3.8 and 27.5/2.7, respectively) and the hedonic ratings were slightly below neutral (Mean/SEM = 37.6/4.5, 40/3.8, 37.4/3.5, respectively).

## Discussion

Axillary odor collected from individuals experiencing various types of stressors has been shown to influence judgments of neutral stimuli (faces) [6] and to differentially activate brain regions [3], whether or not it is discriminable on the basis of odor from non-stress axillary secretions collected from the same individuals [3]. In this study, we evaluated whether smelling untreated axillary secretion collected during a stress manipulation (the Trier Social Stress test) would influence judgments of women portrayed in various everyday situations (e.g., at work, at home, during childcare) relative to axillary secretions collected during exercise or from stress secretions when treated with an unscented anti-perspirant product. Our results suggest that smelling the axillary odors associated with a stress response does negatively influence both dimensions of social evaluations (warmth, competence) that play important roles in a multitude of social interactions. This effect does not appear to be due to stronger or more unpleasant odor from stress sweat, as intensity and pleasantness ratings of the untreated stress samples did not differ from those of exercise or treated stress sweat. To our surprise, however, the negative influence of a stress chemosignal appears to be limited to the social judgments made by males but not females, despite the fact that both genders rated the women as equally stressed when smelling the untreated stress sweat.

We were motivated to understand the basis for the gender difference in chemosensory-mediated social evaluations. The prevailing view that the human stress response for both males and females is characterized by ‘fight or flight’ behavior was challenged by a seminal publication in 2000 [21]. In that review the authors advanced the idea that although those behaviors might comprise the primary physiological response to stress for both men and women, females’ behavioral responses were marked to a greater degree by a pattern of nurturing and social affiliation (‘tend and befriend’). If emotional signals in human sweat can elicit those same emotions in the receiver, as was reliably demonstrated in the de Groot et al study [13], then it is also a reasonable hypothesis that smelling stress sweat can induce some degree of stress in our evaluators. Inhaling the body odors from males in anxiety states was previously shown to be capable of inducing anxiety in a group of female subjects [12]. Following that reasoning, the failure to make negative social judgments of other females, even while rating them as stressed, is consistent with a larger literature showing a strong tendency among both human and non-human females to affiliate with a social group in response to stress and HPA activation [21], [22].

We were also somewhat surprised that the judgments made in the presence of exercise sweat were not statistically different from those made in the presence of untreated stress sweat. As proposed by other researchers [3], [6], we considered sweat obtained from exercise to be an acceptable control for stress sweat. We originally hypothesized that ratings made while smelling sweat obtained from the exercise condition would be significantly higher than those made while smelling the untreated stress sweat. This difference did not reach significance, although average ratings made of women in the exercise sweat condition were more positive than ratings of women in the untreated stress condition. We considered several possible reasons for the failure to see a difference. First, although the apocrine glands are known to enter a refractory period for secretions following a stress challenge [19] (& G. Preti, personal observation), it is possible that residual chemosignals of stress were available and secreted during the exercise challenge that occurred 30 minutes after the TSST ended. Thus, collecting exercise sweat on a different day than the stress sweat collection might have enhanced the difference between them. However, exercising to a target heart rate in the context of a laboratory experiment could also be experienced as a psychosocial stressor if the participant perceived they were being evaluated on their performance. Thus, our samples of exercise sweat could have been contaminated either by residual or new stress secretions and consequently be more similar in composition and impact to the untreated stress sweat samples. In future work, obtaining exercise sweat during a separate session may be a more appropriate control than collecting both types of samples within the same session, provided that the exercise sweat is collected once the participant is familiar with the experimental context and the experimenter does not observe in the presence of the subject.

Overall, the most significant differences were found when comparing ratings made in the presence of sweat from the untreated axilla to those made in the presence of sweat collected from the treated axilla, where a commercial, unscented anti-perspirant product was used to see if it could effectively block the stress sweat signal. The ability of chemosensory signals of psychosocial stress to influence social judgments of other individuals has important implications for both professional and personal interactions and relationships. To be sure, the artificial interactions imposed by our experimental protocol (i.e. rating women in non-verbal videos) may not translate completely to in-person interactions, as other facets of verbal communication may have the potential to overshadow chemosensory signals. However, as a distal sense capable of communicating information across space, it is also likely that airborne communication of stress signals can bias or otherwise predispose an individual to interpret other forms of interactions negatively. Given the preponderance of evidence that chemosignals are processed without conscious awareness [23], recipients of those signals may lack the ability to counteract or modulate the influence of unconscious bias [24]. As our knowledge of the breadth of information that can be conveyed by human body odors expands, it will be important to understand the degree to which such non-verbal communication channels can impact our impression formation in everyday social interactions.
